# Hepcidin and GDF-15 are potential biomarkers of iron deficiency anaemia in chronic kidney disease patients in South Africa

**DOI:** 10.1186/s12882-020-02046-7

**Published:** 2020-09-29

**Authors:** Aishatu M. Nalado, Gbenga Olorunfemi, Therese Dix-Peek, Caroline Dickens, Lungile Khambule, Tracy Snyman, Graham Paget, Johnny Mahlangu, Raquel Duarte, Jaya George, Saraladevi Naicker

**Affiliations:** 1 Department of Internal Medicine, School of Clinical Medicine, Faculty of Health Science, University of the Witwatersrand, 7 York Road, Parktown, Johannesburg, 2193 South Africa; 2 Department of Internal Medicine, College of Health Sciences, Bayero University, Kano, Nigeria; 3 Division of Epidemiology and Biostatistics, School of Public Health, University of the Witwatersrand, Johannesburg, South Africa; 4 Department of Chemical Pathology, National Health Laboratory Services, and School of Pathology, Faculty of Health Sciences, University of Witwatersrand, Johannesburg, South Africa; 5 School of Pathology, Faculty of Health Sciences, University of the Witwatersrand, Johannesburg, South Africa

**Keywords:** Absolute iron deficiency, Chronic kidney disease, Diagnostic test, Functional iron deficiency, GDF-15, Hepcidin, Iron deficiency anaemia, South Africa, Utility, Validity test

## Abstract

**Background:**

Anaemia is a common presenting feature among patients with chronic kidney disease (CKD) and it is associated with poor clinical outcomes and quality of life. It is not clear if growth differentiation factor-15 (GDF-15) or hepcidin are useful as early markers of iron deficiency anaemia (IDA) among non-dialysis CKD patients. We therefore evaluated the diagnostic validity of GDF-15 and hepcidin as biomarkers of IDA among non-dialysis CKD patients in Johannesburg, South Africa.

**Method:**

An analytic cross-sectional study was conducted among non-dialysis CKD patients (*n* = 312) and apparently healthy controls (*n* = 184) from June to December 2016 at an Academic Hospital, in Johannesburg, South Africa. An interviewer administered proforma was used to obtain the socio-biological and clinical characteristics of the participants. Serum levels of GDF-15 and hepcidin were determined. Predictive logistic regression models were built and post estimation receiver operator characteristics were determined to evaluate diagnostic validity of hepcidin and GDF-15 for absolute and functional iron deficiency anaemia.

**Results:**

About half (50.6%) of the participants were female while the participants’ mean age was 49.7 ± 15.8 years. The predictive value of diagnosing absolute IDA among CKD patients using GDF-15 was 74.02% (95% CI: 67.62–80.42%) while the predictive value of diagnosing functional IDA among CKD patients using hepcidin was 70.1% (95% CI: 62.79–77.49%).There was a weak negative correlation between hepcidin levels and GFR (r = − 0.19, *p* = 0.04) in anaemic CKD patients, and between serum GDF-15 and haemoglobin (r = − 0.34, *p* = 0.001). Serum ferritin (β = 0.00389, *P*-value< 0.001), was a predictor of log hepcidin. MCHC (β = − 0.0220, *P*-value 0.005) and CKD stage (β = 0.4761, P-value < 0.001), race (β = 0.3429, P-value = 0.018) were predictors of log GDF-15. Both GDF-15 (adj OR: 1.0003, 95%CI: 1.0001–1.0005, *P* = 0.017) and hepcidin (adj OR: 1.003, 95%CI: 1.0004–1.0055, *P* = 0.023) were associated with iron deficiency anaemia after multiple linear regression modelling.

**Conclusion:**

Serum GDF-15 is a potential biomarker of absolute IDA, while hepcidin levels can predict functional IDA among CKD patients.

## Background

Anaemia is a common presenting feature among patients with chronic kidney disease (CKD) and is associated with poor quality of life and attendant poor clinical outcomes [1]. The pathogenetic mechanisms of anaemia in CKD include reduced erythropoietin production and reticuloendothelial iron blockade, secondary to chronic kidney inflammation [2].

Hepcidin is a peptide hormone that regulates iron balance in the body [3]. It is synthesised by hepatocytes and reduces iron absorption and cellular release of iron, through binding to ferroportin [4]. Thus, in chronic diseases, elevated levels of hepcidin can decrease absorption of dietary iron, impair the release of iron from hepatocytes and macrophages and decrease plasma iron [5]. Studies have also shown that patients with chronic infections and inflammatory disease are predisposed to have increased levels of hepcidin [4, 6]. Elevated hepcidin levels may play a role in the anaemia of chronic kidney disease, as chronic infections and inflammatory disease have been associated with high hepcidin levels [3, 4, 6]. Ideally, hepcidin synthesis should be downregulated when the iron level is low. Nonetheless, inflammatory conditions negatively impact the usual homeostasis of hepcidin, thereby giving room for interleukin-6 to increase hepcidin production even when the iron level is low [7]. Moreover, higher levels of hepcidin may occur among CKD patients, since they are likely to have poor renal clearance of plasma hepcidin, as their kidney function declines [2]. It is also plausible that increased hepcidin concentrations may cause iron-restricted erythropoiesis in CKD-associated anaemia [8]. Hepcidin inhibitors or hepcidin lowering agents [9] have shown promise as adjunctive or stand-alone drugs in the treatment of anaemia of inflammation in pre-clinical studies [10]. From these previous observations, we postulate that the serum hepcidin level may be a useful biomarker of iron status among CKD patients. However, the utility of hepcidin as a biomarker in the diagnosis of iron deficiency anaemia (IDA) among non-dialysis CKD patients is unclear, especially in low resource settings such as ours.

Growth Differentiation Factor-15 (GDF-15), an anti-inflammatory cytokine, has been suggested as a significant regulator of hepcidin [11]. Oxidative stress and inflammatory conditions can stimulate secretion of GDF-15 by macrophages [12]. In addition, a strong positive correlation has been reported between hepcidin and GDF-15 in anaemic patients [13]. Emerging evidence shows that in response to anaemia [14], erythroblasts secrete GDF-15, which in turn suppresses hepcidin expression and decreases iron stores. In addition, a strong positive correlation has been reported between hepcidin and GDF-15 in anaemic patients [13]. Emerging evidence shows that in response to anaemia [14], erythroblasts secrete GDF-15, which in turn suppresses hepcidin expression and decreases iron stores [12, 15, 16]. The role of GDF-15 in the evolution of IDA is still controversial. However, studies demonstrating a relationship (or otherwise) between GDF-15 expression and serum iron parameters among patients with IDA will assist to shed more light on the investigation, treatment and monitoring of IDA in CKD patients [17].

Therefore, the aim of this study was to evaluate serum GDF-15 and hepcidin as surrogates or diagnostic markers of IDA among non-dialysis requiring CKD patients.

## Methods

We conducted a comparative cross-sectional study among 312 consecutive patients with CKD attending the renal outpatient clinic of Charlotte Maxeke Johannesburg Academic Hospital (CMJAH), South Africa from 1 June 2016 to 31 December 2016, and 184 healthy controls (which included patients’ relatives and staff members). The inclusion criterion was all adult stable consenting non-dialysis CKD patients aged 18 years and above. CKD patients attending nephrology clinics of CMJAH, that were diagnosed based on estimated glomerular filtration rate (eGFR) < 60 ml/min were approached during their clinic visits to participate in the study. Apparently, healthy consenting controls who were hospital staff and relations of patients were approached and recruited as controls. We evaluated each prospective control for any history of acute or chronic medical condition. Only those participants with no known history of ill health were recruited as controls for the study. Venous samples for C-reactive protein was obtained from the participants. Active inflammation and infection were suspected if the C-reactive protein was > 5 mg/l and/or there was a clinical history suggestive of inflammatory and/or infective process.

The Human Research Ethics Committee at the University of the Witwatersrand approved this study (clearance certificate number 150929). All participants gave written informed consent.

Exclusion criteria included active inflammation, active infection, haemoglobinopathies, and blood transfusion within 3 months preceding the study, immunosuppressive therapy, oral iron treatment, and use of intravenous iron therapy at least 2 weeks before enrolment, and persons currently on erythropoietin stimulating agents (ESA).

We defined absolute iron deficiency as serum ferritin <100μg/l and transferrin saturation (TSAT < 20%), while functional iron deficiency was defined as serum ferritin >100μg/l and TSAT < 20%, according to Capellini et al. [18]. Anaemia was defined as haemoglobin < 13 g/l in men and < 12 g/l in women. Patients were considered to have IDA if they presented with absolute iron deficiency, functional iron deficiency and anaemia with low mean corpuscular volume (MCV) [19, 20]. Glomerular filtration rate (GFR) was determined by the Chronic Kidney Disease Epidemiology Collaboration (CKD-EPI) equation for eGFR [21].

### Laboratory measurements

All collected venous blood samples were immediately centrifuged, separated into aliquots, and stored at − 80 degrees Celsius for future assays. Haematological measurements were made on fresh venous blood with EDTA and clotted blood samples. Haemoglobin concentrations (Hb), red blood cell count (RBC), platelet count, creatinine and urea were measured using a haematology analyser (Siemens Diagnostics, Tarrytown, USA). We determined the serum iron total iron-binding capacity (TIBC) and ferritin as previously described [22]. Transferrin saturation (TSAT) was calculated as the ratio of serum iron and TIBC and expressed as a percentage.

Hepcidin and isotope labelled internal standards [^13^C_9_,^15^N_3_] were purchased from Peptide Institute (Osaka JPN). The serum hepcidin-25 and isotope labelled internal standards [^13^C_9_,^15^N_3_] were extracted and separated as previously described by Li et al. [23] Briefly, samples were extracted with 4% HPLC grade trichloroacetic acid (TCA, Merck, Kenilworth, NJ, U.S.A) and separated using reverse-phase liquid chromatography using a 0.5x50mm Halo Peptide- ES column (Sciex, Framingham, USA) with a total run time of 5 min per sample. The analysis was performed on an AB Sciex 5500 QTRAP (Sciex, Framingham, USA), coupled to a micro-Liquid Chromatography (Exigent M3) system with Turbo Ion Sprayionisation source operated in positive ion mode. Multiple reactive monitoring (MRM) detection was applied using the Sciex 5500 for identification and quantification using the ion transitions (m/z) of 698.0 > 354.0 and 703.2 > 354.1 for hepcidin and stable isotope labelled hepcidin [^13^C_9_,^15^N_3_] respectively.

Concentrations of serum hepcidin were expressed in nanograms per millilitre (ng/ml). The intra-and inter-assay coefficients of variation (CV) were < 6.7 and < 8.8%, respectively. The lower limit of detection was 0.5 ng/ml. The median reference level for plasma hepcidin in healthy controls was 5.7 ng/ml. Serum levels of GDF-15 were measured by ELISA (R&D Systems, Minneapolis, MN, USA) with intra- and inter-assay CVs of < 2.8 and < 6, respectively.

### Statistical analysis

Statistical analysis was performed using Stata version 14 (Stata Corp., TX, USA) software. Descriptive statistics were conducted. The categorical variables were described as frequency and percentages and skewness Kurtosis test was used to determine normality. Subsequently, continuous variables were described as mean (± standard deviation), if normally distributed or as median (interquartile range) when not normally distributed. The 5th and 95th percentile of the hepcidin and GDF-15 levels among non-anaemic apparently healthy controls were determined as the reference range for the study population. Student’s t-test or Mann-Whitney U tests were used to compare continuous variables among anaemic and non-anaemic groups. Association between anaemic status and categorical variables were assessed using Pearson’s Chi-square test. Spearman’s rank correlation was used to determine the linear relationship between hepcidin or GDF-15 and other linear variables among anaemic and non-anaemic participants. Simple and multiple linear regression was conducted with log hepcidin and logGDF-15 as outcome variables respectively. Univariable and multivariable logistic regression was conducted to determine the association between IDA and hepcidin. Confounding variables based on the literature and a univariable *p*-value of < 0.2 were utilised to build the multivariable model using backwards stepwise regression. Age and gender were chosen a priori. A post-estimation receiver operator characteristic curve (ROC) with an area under the curve (AUC) was then utilised to determine the predictive value of hepcidin for IDA among CKD participants. Similar regression analysis was conducted between IDA and GDF-15. The AUC of ROC for hepcidin and GDF-15 were then compared. A non-covariate table of cut-offs with corresponding sensitivity and specificity was generated for hepcidin as a diagnostic marker for IDA among the CKD population. The optimum cut-off point of hepcidin was then calculated based on the maximal value of the Younden Index (= (sensitivity + specificity) -1). Similarly, an optimum cut-off value for GDF-15 was obtained. The above-mentioned analysis (regression, ROC and cut-offs) was conducted to determine the predictive value of hepcidin and then GDF-15 for functional and absolute IDA. Sub-group analysis was conducted to see if there was a disparity in the performance of hepcidin and GDF-15 in the diagnosis of absolute and functional IDA. The predictive/ diagnostic value of hepcidin or GDF-15 was stratified across the stages (early [I-III], late [IV-V]) and common aetiologies of CKD. For all analyses, a 2-tailed test of the hypothesis was assumed and the level of statistical significance was set at *P*-value < 0.05 (95% confidence interval).

## Results

The mean age of the participants was 49.7 (± 15.8) years and there was an almost equal proportion of male (49.4%, *n* = 245/496) and female (50.6%, *n* = 251/496) participants. The prevalence of anaemia among CKD cases was about three times that of controls, 33.0% (95%CI: 27.99–38.45%) vs 9.78% (95%CI: 6.22 - 15.05%) respectively (Table 1).

**Table 1:** Socio-demographic, haematological and biochemical characteristics of participants by iron-deficiency-anaemia status

Characteristics	CKD (***n*** = 312)			Controls (***n*** = 184)		
	IDA ***n*** = 103 (%)	Non-anaemic ***n*** = 209 (%)	***P***-value	IDA ***n*** = 18 (%)	Non-anaemic ***n*** = 166 (%)	***P***-value
Hepcidin (ng/ml) (median, IQR)	8.4 (4–45.5)	6.8 (3.9–33.6)	0.2790	3.1 (2.3–7.9)	3.1 (2.1–10.9)	0.836
GDF-15 (pg/ml) (median, IQR)	1256.8 (919.1–1618)	700 (335.1–1327.8)	< 0.0001	1175.9 (708.9–1267.1)	397.95 (183.2–1101)	0.016
**Age (mean ± SD) years**	54.5 ± 15.2	54.3 ± 14.2	0.9340	45.2 ± 17.5	41.3 ± 13.6	0.255
< 40	15 (14.6)	37 (17.7)	0.484	8 (44.4)	79 (47.6)	0.800
≥40	88 (82.3)	172 (85.4)		10 (55.6)	87 (52.4)	
**Race**						
Blacks	95 (92.2)	165 (79.0)	0.003	12 (66.7)	133 (80.1)	0.185
Whites	8 (7.8)	44 (21.1)		6 (33.3)	33 (19.9)	
**Gender**						
Male	41 (39.8)	126 (60.3)	0.001	7 (38.9)	71 (42.8)	0.752
Female	62 (60.2)	83 (39.7)		11 (61.1)	95 (57.2)	
**Systolic Blood Pressure (median, IQR) (mmHg)**	139 (125–157)	140 (130–160)	0.1994	132 (120–140)	132 (130–140)	0.998
**Diastolic Blood Pressure (median, IQR) (mmHg)**	80 (70–91)	80 (70–90)	0.6234	81 (70–90)	80 (72–90)	0.7707
**Serum Urea (median, IQR)** **(mmol/L)**	17.1 (9.8–25.7)	11 (7.1–15.9)	0.0001	4 (3–4.9)	4.35 (3.6–5.2)	0.4486
**Serum Creatinine** **(median, IQR),** **μmol/L**	265 (158–520)	171 (120–255)	< 0.0001	70.5 (64–78)	78.5 69–91)	< 0.0001
**eGFR (median, IQR), mls/min/1.73m**^**2**^	27.0 (12.6–27.0)	43.6 (27.7–66.0	< 0.0001	129.8 (96.3–139.5)	114.4 (96.8–133.0)	0.3490

Table 1 also showed that the median levels of serum GDF-15 [1024.5 (429.4–1489.5 pg/ml) vs. 447.25 (188.25–1192.3 pg/ml), *P*-value< 0.0001] and hepcidin [7.1 (3.9–36.2) vs 3.1(2.1–10.9), P-value < 0.0001], were more than doubled among the CKD participants as compared to the controls. Among the CKD participants, the median GDF-15 level was higher among the anaemic as compared to the non-anaemic participants (*P*-value < 0.0001, Table 1). Similarly, median GDF-15 levels were higher among the anaemic controls as compared to the non-anaemic controls (*P* = 0.0155). In contrast, there was no difference in the serum levels of hepcidin by anaemia status among the CKD participants (P-value =0.2790) and the controls (P-value = 0.8357).

The reference values (5–95% range) among the non-anaemic controls of this study for hepcidin and GDF-15 were 1.5–28.1 ng/ml and 108.2–2833 pg/ml, respectively. Furthermore, the reference range (5th – 95th percentile) of hepcidin among apparently healthy female participants was 1.4–38.8 ng/ml while the reference range for males was 1.6–28.1 ng/ml. There was a significant negative linear relationship between hepcidin and GDF-15 among anaemic CKD patients (*r* = − 0.28, *P*-value = 0.0037), supplementary Table 1.

Serum ferritin (*r* = 0.5, *P*-value < 0.0001), and serum creatinine levels (*r* = 0.21, *P*-value = 0.0368) were positively correlated with hepcidin, while GFR (*r* = − 0.19, *P*-value = 0.0493) was negatively correlated with hepcidin among the participants with CKD, supplementary Table 1. GDF-15 was negatively correlated with serum ferritin and haemoglobin levels, while TSAT level correlated with GDF-15, especially among the anaemic CKD cases (supplementary Table 2).

For every ng/ml increase in serum ferritin level among CKD patients, log hepcidin increased by 0.00389 (β = 0.00389, *P*-value< 0.001). In other words, hepcidin levels increased by 10^0.00389^ units for every ng/ml increase in serum ferritin.

logGDF-15 decreased with every unit increase of MCHC (β = − 0.0618, *P*-value = 0.005). On average, the white racial group and those participants with late CKD stage (IV-V) had a higher log GDF-15 as compared to the black racial group (β = 0.343, *P*-value = 0.018) and early CKD stage (I-III) (β = 0.476, *P*-value < 0.001); Table 2.

**Table 2:** Multiple linear predictors of log Hepcidin and logGDF-15 among CKD patients

Variable	Log Hepcidin			Log GDF-15		
	Coefficient	SE	***P***-value	Coefficient	SE	***P***-value
Ferritin	0.00389	0.00064	< 0.0001	−0.00038	0.00039	0.328
Age	0.0017	0.00581	0.766	0.0016	0.0036	0.648
Race						
Blacks	Reference	Reference	Reference	Reference	Reference	Reference
Whites	−0.2301	0.2239	0.303	0.3429	0.1443	0.018
Gender						
Male	Reference	Reference	Reference	Reference	Reference	Reference
Female	−0.2336	0.1572	0.138	0.1688	0.1041	0.106
MCHC	−0.0393	0.0376	0.298	−0.0618	0.0220	0.005
MCV	0.0082	0.0124	0.506	−0.0136	0.0073	0.066
CKD Stage						
Early(I-III)	Reference	Reference	Reference	Reference	Reference	Reference
Late (IV – V)	−0.0223	0.1656	0.893	0.4761	0.0993	< 0.0001

**Fig. 1:**
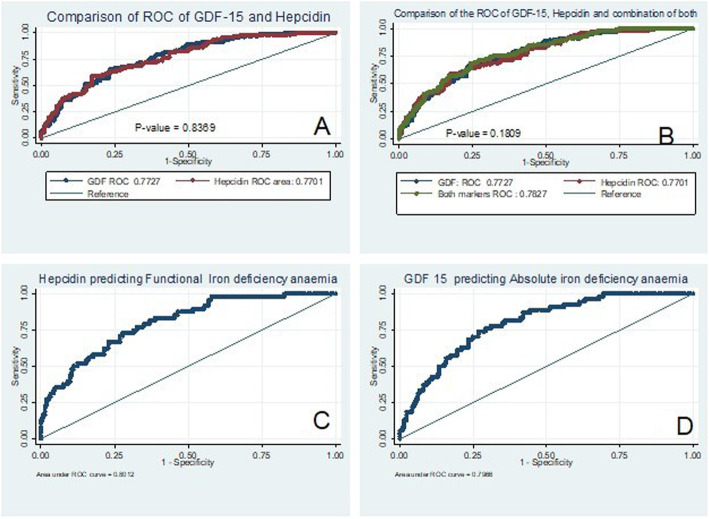
Receiver Operator characteristics curves of: **a**. Comparison of the predictive value of Hepcidin and GDF-15 in diagnosing Iron deficiency anaemia, **b**. Comparison of the predictive value of Hepcidin and GDF-15 and combination of both markers in diagnosing Iron deficiency anaemia. **c**. Predictive value of Hepcidin in diagnosing functional Iron deficency anaemia. **d**. Predictive value of GDF-15 in diagnosing absolute Iron deficiency anaemia

The predictive value of GDF-15 and hepcidin for diagnosing IDA among CKD participants was 77.27% (95%CI: 71.98–82.56%) and 77.01% (95%CI: 71.64–82.38%), respectively (Table 3, Fig. 1a). There was no statistically significant difference between the AUC of the ROC curves of GDF-15 and that of hepcidin (*P*-value = 0.8369); (Fig. 1a). A combination of the two parameters did not improve the diagnostic value of either of the two tests, as the AUC of the ROC of the model with the combination was 78.27, 95%CI: 73.08–83.47%; *P*-value = 0.1809 Fig. 1b.

**Table 3:** Relationship between iron deficiency anaemia and Hepcidin or GDF-15 as primary biomarker among CKD participants

Variable	^**a**^**Multivariable logistic regression analysis with Hepcidin as the primary factor**			^**b**^**Multivariable logistic regression analysis with GFD15 as the primary factor**		
	Adjusted Odds ratio	95%CI	***P***-value	Adjusted Odds ratio	95%CI	***P***-value
**Hepcidin**	1.0030	1.0004–1.0055	0.023	–	–	–
**GDF-15**		–	–	1.0003	1.0001–1.0005	0.017

^**a**^Multivariable logistic regression of the model of the relationship between Hepcidin and iron deficiency anaemia

^**b**^Multivariable logistic regression of model of the relationship between GDF-15 and iron deficiency anaemia

The 2 models corrected for gender, age, CKD stage, history of diabetes mellitus, race, C-reactive protein, mean corpuscular volume

*CI* Confidence interval

Using the non-covariate analysis and Younden’s index, the optimum cut-off value among CKD participants for GDF-15 was 1030 pg/ml (at a sensitivity of 72.8% and specificity of 61.24%). Similarly, the optimum cut-off for hepcidin was 22.5 ng/ml (at a sensitivity of 38.8% and specificity of 70.8%); (Data not shown).

The predictive value of hepcidin for diagnosing functional IDA among CKD participants was 80.12%. (Table 4, Fig. 1c). The predictive value of GDF-15 for diagnosing absolute IDA among CKD participants was 79.66%. (Table 5, Fig. 1d). Using the non-covariate analysis and Younden’s index, the optimum cut-off value of GDF-15 for diagnosing absolute IDA among CKD participants was 1129.3 mg/dl (at a sensitivity of 83.64%and specificity of 66.03%) Similarly, the optimum cut-off for hepcidin was 22.5 ng/dl (at a sensitivity of 66.7% and specificity of 70.8%); (Supplementary Table 3).

**Table 4:** Relationship between functional iron deficiency anaemia and Hepcidin or GDF-15 as primary biomarker among CKD participants

Variable	^**a**^**Multivariable logistic regression analysis with Hepcidin as the primary factor**			^**b**^**Multivariable logistic regression analysis with GFD15 as the primary factor**		
	Adjusted Odds ratio	95%CI	***P***-value	Adjusted Odds ratio	95%CI	***P***-value
**Hepcidin**	1.0043	1.00041–1.00829	0.030	–	–	–
**GDF-15**	–	–	–	1.00007	0.99970–1.00044	0.715

^a^Multivariable logistic regression of the model of the relationship between Hepcidin and iron deficiency anaemia

^b^Multivariable logistic regression of model of the relationship between GDF-15 and iron deficiency anaemia

The 2 models corrected for gender, age, CKD stage, history of Diabetes mellitus, race, C-reactive protein, mean corpuscular volume

*CI* Confidence interval

**Table 5:** Relationship between absolute iron deficiency anaemia and Hepcidin or GDF-15 as primary biomarker among CKD participants

Variable	^**a**^**Multivariable logistic regression analysis with Hepcidin as the primary factor**			^**b**^**Multivariable logistic regression analysis with GFD15 as the primary factor**		
	Adjusted Odds ratio	95%CI	***P***-value	Adjusted Odds ratio	95%CI	***P***-value
**Hepcidin**	1.0021	0.9991–1.0051	0.176	–	–	–
**GDF-15**	–	–	–	1.00038	1.0001–1.0006	0.003

^a^Multivariable logistic regression of the model of the relationship between Hepcidin and iron deficiency anaemia

^b^Multivariable logistic regression of model of the relationship between GDF-15 and iron deficiency anaemia

The 2 models corrected for gender, age, CKD stage history of Diabetes mellitus, race, C-reactive protein, mean corpuscular volume

*CI* Confidence interval

From Supplementary Table 4, we found that although median serum hepcidin level among participants with late stage CKD (IV – V) was more than double the median hepcidin level among participants with early disease (I-III), this relationship did not reach statistical significance among ID ((Early Vs Late, 4.7(3.9–32.55) Vs 10.1 (4.1–55.8), *P* = 0.2583)) and non ID ((6.2(3.9–21.6) Vs 14.3(4–48), *P* = 0.1387)) participants. Median GDF-15 levels were also higher among participants with late stage as compared to participants with early stage disease. There was no statistically significant difference in the median hepcidin and median GDF-15 levels across the different aetiologies of CKD.

Further sub-analysis showed that hepcidin was not statistically associated with AID and ID among both early and late stage disease. However, the predictive value of diagnosing FID among late stage CKD disease using hepcidin as a biomarker was about 75.2%, while there was no statistically significant association between hepcidin and FID among patients with early stage CKD; (Supplementary Table 5).

GDF 15 was not associated with FID among both early and late CKD patients. However, the predictive value of GDF-15 for diagnosing AID and ID among early stage disease was about 83.3 and 77.5% respectively. Whereas, there was no statistically significant relationship among GDF and AID or ID among the late stage CKD participants; (Supplementary Table 5).

Among patients with a diagnosis of hypertension, GDF-15 predicted AID and ID anaemia in 81.1 and 81.9% of cases respectively; (Supplementary Table 5).

## Discussion

To our knowledge, this is the first study to evaluate the clinical utility of hepcidin and GDF-15 as markers of IDA, among persons with non-dialysis CKD. This study demonstrates that in a cohort of predialysis CKD patients, serum hepcidin and GDF-15 predicted IDA among patients with CKD, with a predictive value of 71.4 and 72.3%, respectively. Furthermore, GDF-15 predicted AID anaemia, while hepcidin predicted FID anaemia (83.6% vs 66.7%). In addition, we showed a negative correlation between hepcidin and eGFR among CKD patients.

Our observation of a negative correlation of hepcidin with GFR agrees with findings by others [8, 24]. However, some authors found a positive correlation between GFR and hepcidin levels [25, 26]. This suggests that the relationship between hepcidin and renal function is still unclear. Moreover, hepcidin pathways may be largely dependent on ferritin metabolism, with renal function playing a minimal role. The smaller sample size of previous studies may affect their conclusions. Our study recruited a relatively larger sample size. The sensitivity of the methods used may also play a role in the observed differences between our findings and others as we used mass spectrometry, in contrast to other studies where ELISA was used [24, 27].

Our data supports published studies reporting higher levels of hepcidin in CKD and among haemodialysis patients [25, 28]. The increase in serum hepcidin levels in CKD patients compared to controls might be due to increased inflammation and decreased renal clearance of hepcidin attributable to renal impairment in CKD patients [29–31].

We found that serum ferritin, the primary storage molecule for cellular iron, positively correlated with hepcidin in our CKD patients as previously documented among patients with CKD and those on dialysis [25, 28, 32–34]. This finding is also consistent with studies in non-CKD populations [35–37]. In the setting of CKD, the direct relationship of hepcidin with ferritin may represent a protective effect of hepcidin against systemic iron overload [26]. However, there was no correlation between hepcidin and inflammatory markers such as hsCRP. The possible explanation may be that patients with active infections and inflammation were excluded from our study.

The reference range (5–95%) of serum hepcidin levels among healthy controls in this study was 1.5–28.1 ng/ml. This value was lower than the reference range reported among healthy male [median and 5–95% reference range, 112 ng/ml (29-254 ng/ml) and female (65 ng/ml (17-286 ng/ml)] adults [32]. Furthermore, the reference range of hepcidin among apparently healthy female participants was 1.4–38.8 ng/ml while the reference range for male participants was 1.6–28.1 ng/ml. These values were lower than the reference range reported among healthy male [median and 5–95% reference range, 112 ng/ml (29-254 ng/ml) and female (65 ng/ml (17-286 ng/ml)] adults [32]. A possible explanation may be differences in assay used and racial differences. Thus, there is a need to establish different reference ranges for diverse populations.

We determined a cut-off value for serum hepcidin of < 22.5 ng/ml, (AUC = 0.71) at a sensitivity of 38.8%, and specificity of 70.8%, to predict IDA, which supports the literature that hepcidin levels are below the reference range in iron-deficient states [38]. Several researchers have reported different cut-offs with corresponding sensitivity and specificity for diagnosing IDA among diverse study populations [39–41]. These findings differ from the study by Jonker et al. [42], who reported hepcidin to be a poor predictor of bone marrow iron stores (sensitivity of 66.7% and specificity 48.5%). However, their study population included children, and intra-individual variability in serum hepcidin could account for differences in our findings. This is the first study that showed in a sub-group analysis that hepcidin was predictive of functional IDA in pre-dialysis CKD. Although levels of hepcidin are elevated in CKD, other factors may affect hepcidin levels. Further research is needed to determine whether this biomarker has advantages over conventional markers (ferritin, TSAT) [43].

We found GDF-15 levels to be significantly higher in CKD patients with IDA as compared to CKD patients without IDA, consistent with findings by other researchers [16, 44]. Although Li et al. [45] found an increase in GDF-15 levels in their dialysis patients, they were unable to demonstrate a correlation between GDF-15 and iron indices, a finding that was similarly noted among our cohort of participants. There are possible mechanisms that may explain the findings of increased GDF-15 in patients with IDA. First, GDF-15 may be an important mediator in a negative feedback loop whereby it increases to suppress high hepcidin levels in CKD patients with iron deficiency. Another explanation is that iron depletion could independently cause GDF-15 induction in the erythroid precursor cells as a result of iron sequestration in macrophages [46]. All these are speculative, however, as the exact mechanism, underlying IDA and GDF-15 levels require further investigation.

We also found that GDF-15 predicted IDA at a cut-off value < 1030 pg/ml with a predictive value of 72% (AUC = 0.723), sensitivity of 72.8%, and specificity of 61.24%. In contrast, Tanno et al. [47] found among blood donors that there was no association between iron deficiency due to blood loss and serum GDF-15 levels. Our study, therefore, suggests that GDF-15 can be a useful diagnostic tool in patients with IDA. However, inflammation-mediated changes seen in iron homoeostasis may not induce the increased GDF-15 levels in patients with IDA, as serum ferritin levels did not correlate with GDF-15 levels in this study and as reported by Mast et al. [48], GDF-15 in this study was found to be predictive of absolute IDA (but not functional IDA) in sub-group analysis. Our report contrasts with the findings of Yilmaz et al. [44], where GDF-15 was predictive of functional IDA among haemodialysis and CKD patients. Differences in these findings could be due to differences in the study population, as their study population included patient on dialysis, while we recruited and studied pre-dialysis patients. Another explanation could be due to different cut-off values to define functional IDA; their cut-off value was > 800, while ours was > 100. Our sub-analysis suggests that GDF can predict AID and ID anaemia in more than 80% of cases among patients with early CKD (stage I-III), but was not shown to predict AID nor ID among participants with late stage kidney disease (Stages IV/V). This report is similar to our previous finding that reticulocyte haemoglobin content had a higher predictive value for diagnosing iron deficiency anaemia among early stage CKD patients as compared to late stage CKD patients (AUC for early Vs late: 0.82 Vs 0.76 [49]. In the meantime, there may be need to develop recommendations on the use of biomarkers as screening tools in CKD patients that would stratify the performance of the test by CKD stage.

Another important finding in this study is the negative correlation of GDF-15 with haemoglobin in both CKD patients and controls. This finding is consistent with reports by others [45, 50, 51], but is in disagreement with findings in non-CKD populations, where GDF-15 was positively correlated with haemoglobin [52–54]. The difference in findings between our study and others could be explained by racial differences and diversity of ethnicity in the studied population.

Our study has limitations. First, the cross-sectional study design makes it difficult to infer causality between hepcidin, GDF-15 levels, and the risk of anaemia. The cross-sectional study design also precluded us from conducting follow up serial measurements of both hepcidin and GDF-15. The intra-patient variability and diurnal variations in hepcidin assays may interfere with serum hepcidin measurement.

The strengths of our study include the large sample size of pre-dialysis CKD patients, and our use of mass spectrometry, the gold standard for hepcidin assay. In addition, our study defined iron deficiency based on absolute and functional IDA, whereas prior studies focused mostly on functional IDA, and mainly recruited dialysis patients. Finally, to our knowledge, this is the first study to predict the performance of GDF-15 as a diagnostic tool for IDA in non-dialysis CKD patients.

## Conclusion

Our preliminary data indicate that GDF-15 and hepcidin predict IDA in pre-dialysis CKD patients and could be promising tools in the diagnosis of IDA in CKD; they could predict absolute and functional IDA among pre-dialysis CKD. These assays are expensive, not readily available, and require technical know-how, which may preclude their use in resource-constrained environments. More extensive randomized prospective studies are necessary to confirm our findings and to help determine a reliable cut-off value of serum hepcidin and GDF-15 in the diagnosis of IDA.

## Supplementary information

**Additional file 1 Table S1:** Correlations of hepcidin levels with haematological and other parameters. **Table S2:** Correlation of GDF-15 levels and iron parameters among the study population. **Table S3:** Cut-off and validity of GDF-15 and Hepcidin in diagnosing Absolute and functional Iron deficiency Anaemia among CKD patients. **Table S4.** Relationship between hepcidin and GDF15 levels across stages and aetiology of Chronic kidney disease. **Table S5.** Performance of Hepcidin and GDF-15 in the diagnosis of iron deficiency anaemia among chronic kidney disease participants across stage and aetiology of kidney disease.

## Data Availability

The dataset used in the analysis is available with the corresponding author and will be released on request.

## Abbreviations

AID: Absolute iron deficiency anaemia
AUC: Area under the curve
CKD: Chronic kidney Disease
CMJAH: Charlotte Maxeke Johannesburg Academic Hospital
CKD-EPI: Chronic Kidney Disease Epidemiology Collaboration
ESA: Erythropoietin stimulating agents
FID: Functional iron deficiency
GDF-15: Growth differentiation factor-15
HB: Haemoglobin
IDA: Iron deficiency anaemia
MCHC: Mean corpuscular haemoglobin concentration
MCV: Mean corpuscular volume
TIBC: Total iron binding capacity
TSAT: Transferrin saturation

## References

[1] Mikhail A, Brown C, Williams JA, Mathrani V, Shrivastava R, Evans J, et al. Renal association clinical practice guideline on Anaemia of chronic kidney disease. BMC Nephrol. 2017;18. 10.1186/s12882-017-0688-1 PMC5709852 29191165

[2] Ganz T (2007). Molecular control of iron transport. J Am Soc Nephrol.

[3] Nemeth E, Tuttle MS, Powelson J, Vaughn MB, Donovan A, Ward DM (2004). Hepcidin regulates cellular iron efflux by binding to ferroportin and inducing its internalization. Science (New York, NY).

[4] Arezes J, Nemeth E (2015). Hepcidin and iron disorders: new biology and clinical approaches. Int J Lab Hematol.

[5] Pak M, Lopez MA, Gabayan V, Ganz T, Rivera S (2006). Suppression of hepcidin during anemia requires erythropoietic activity. Blood..

[6] Cheng PP, Jiao XY, Wang XH, Lin JH, Cai YM (2011). Hepcidin expression in anemia of chronic disease and concomitant iron-deficiency anemia. Clin Exp Med.

[7] D'angelo G (2013). Role of hepcidin in the pathophysiology and diagnosis of anemia. Blood Res.

[8] Uehata T, Tomosugi N, Shoji T, Sakaguchi Y, Suzuki A, Kaneko T (2012). Serum hepcidin-25 levels and anemia in non-dialysis chronic kidney disease patients: a cross-sectional study. Nephrology Dialysis Transplantation.

[9] Tsuchiya K, Nitta K (2013). Hepcidin is a potential regulator of iron status in chronic kidney disease. Ther Apheresis Dial.

[10] Wang C-Y, Babitt JL (2016). Hepcidin regulation in the anemia of inflammation. Curr Opin Hematol.

[11] Yalcin MM, Altinova AE, Akturk M, Gulbahar O, Arslan E, Ors Sendogan D, et al. GDF-15 and Hepcidin levels in nonanemic patients with impaired glucose tolerance. J Diabetes Res. 2016. 10.1155/2016/1240843. 10.1155/2016/1240843 PMC5014962 27642607

[12] Bootcov MR, Bauskin AR, Valenzuela SM, Moore AG, Bansal M, He XY (1997). MIC-1, a novel macrophage inhibitory cytokine, is a divergent member of the TGF-beta superfamily. Proc Natl Acad Sci U S A.

[13] Mehmet Muhittin Yalcin AEA, Akturk M (2016). GDF-15 and Hepcidin levels in nonanemic patients with impaired glucose tolerance. J Diabetes Res.

[14] Valenti L, Messa P, Pelusi S, Campostrini N, Girelli D (2014). Hepcidin levels in chronic hemodialysis patients: a critical evaluation. Clin Chem Lab Med.

[15] Tanno T, Miller JL (2011). [GDF15 expression and iron overload in ineffective erythropoiesis]. [Rinsho ketsueki]. Japanese J Clin Hematol.

[16] Lakhal S, Talbot NP, Crosby A, Stoepker C, Townsend AR, Robbins PA (2009). Regulation of growth differentiation factor 15 expression by intracellular iron. Blood..

[17] Abaza HM, Habashy DM, El-Nashar RE (2013). Growth differentiation factor 15 expression in anemia of chronic disease and iron deficiency anemia. Egyptian J Haematology.

[18] Cappellini MD, Comin-Colet J, de Francisco A, Dignass A, Doehner W, Lam CS (2017). Iron deficiency across chronic inflammatory conditions: international expert opinion on definition, diagnosis, and management. Am J Hematol.

[19] Wish JB (2006). Assessing iron status: beyond serum ferritin and transferrin saturation. Clin J Am Soc Nephrol..

[20] Stauffer ME, Fan T (2014). Prevalence of Anemia in chronic kidney disease in the United States. PLoS One.

[21] van den Brand JA, van Boekel GA, Willems HL, Kiemeney LA, den Heijer M, Wetzels JF (2011). Introduction of the CKD-EPI equation to estimate glomerular filtration rate in a Caucasian population. Nephrology, dialysis, transplantation : official publication of the European Dialysis and Transplant Association - European Renal Association..

[22] Nalado AM, Mahlangu JN, Waziri B, Duarte R, Paget G, Olorunfemi G (2019). Ethnic prevalence of anemia and predictors of anemia among chronic kidney disease patients at a tertiary hospital in Johannesburg, South Africa. Int J Nephrol Renovascular Dis.

[23] Li H, Rose MJ, Tran L, Zhang J, Miranda LP, James CA (2009). Development of a method for the sensitive and quantitative determination of hepcidin in human serum using LC-MS/MS. J Pharmacol Toxicol Methods.

[24] Peters HP, Laarakkers CM, Swinkels DW, Wetzels JF (2010). Serum hepcidin-25 levels in patients with chronic kidney disease are independent of glomerular filtration rate. Nephrology Dialysis Transpl.

[25] Ashby DR, Gale DP, Busbridge M, Murphy KG, Duncan ND, Cairns TD (2009). Plasma hepcidin levels are elevated but responsive to erythropoietin therapy in renal disease. Kidney Int.

[26] Zaritsky J, Young B, Wang HJ, Westerman M, Olbina G, Nemeth E (2009). Hepcidin—a potential novel biomarker for Iron status in chronic kidney disease. Clin J Am Soc Nephrology.

[27] van der Putten K, Jie KE, van den Broek D, Kraaijenhagen RJ, Laarakkers C, Swinkels DW (2010). Hepcidin-25 is a marker of the response rather than resistance to exogenous erythropoietin in chronic kidney disease/chronic heart failure patients. Eur J Heart Fail.

[28] Tomosugi N, Kawabata H, Wakatabe R, Higuchi M, Yamaya H, Umehara H (2006). Detection of serum hepcidin in renal failure and inflammation by using ProteinChip system. Blood..

[29] Kulaksiz H, Gehrke SG, Janetzko A, Rost D, Bruckner T, Kallinowski B (2004). Pro-hepcidin: expression and cell specific localisation in the liver and its regulation in hereditary haemochromatosis, chronic renal insufficiency, and renal anaemia. Gut..

[30] van der Weerd NC, Grooteman MP, Bots ML, van den Dorpel MA, den Hoedt CH, Mazairac AH (2012). Hepcidin-25 in Chronic Hemodialysis Patients Is Related to Residual Kidney Function and Not to Treatment with Erythropoiesis Stimulating Agents. PLos One.

[31] Nicolas G, Chauvet C, Viatte L, Danan JL, Bigard X, Devaux I (2002). The gene encoding the iron regulatory peptide hepcidin is regulated by anemia, hypoxia, and inflammation. J Clin Invest.

[32] Ganz T, Olbina G, Girelli D, Nemeth E, Westerman M (2008). Immunoassay for human serum hepcidin. Blood..

[33] JFM WDWS (2008). Hepcidin: a new tool in the management of anaemia in patients with chronic kidney disease?. Nephrology Dialysis Transpl.

[34] Coyne DW (2011). Hepcidin: clinical utility as a diagnostic tool and therapeutic target. Kidney Int.

[35] Ingo Mecklenburg DR, Reznik D, Fasler-Kan E, Drewe J, Beglinger C, Hruz P (2014). Serum hepcidin concentrations correlate with ferritin in patients with inflammatory bowel disease. J Crohns Colitis.

[36] Haghpanah S, Esmaeilzadeh M, Honar N, Hassani F, Dehbozorgian J, Rezaei N (2015). Relationship Between Serum Hepcidin and Ferritin Levels in Patients With Thalassemia Major and Intermedia in Southern Iran. Iran Red Crescent Med J.

[37] BPE FAN, Ruiz MA (2014). Assessmet of Labile Plasma Iron and Hepcidin in Patients Who Undergo Hematopoietic Stem Cell Transplantation. Blood.

[38] Goyal J, McCleskey B, Adamski J (2013). Peering into the future: hepcidin testing. Am J Hematol.

[39] Pasricha SR, McQuilten Z, Westerman M, Keller A, Nemeth E, Ganz T (2011). Serum hepcidin as a diagnostic test of iron deficiency in premenopausal female blood donors. Haematologica..

[40] Vyas S, Kapoor A, Nema S, Suman S (2017). Quantification of serum hepcidin as a potential biomarker in diagnosis of iron deficiency anaemia. Int J Res Med Sci.

[41] Choi HS, Song SH, Lee JH, Kim HJ, Yang HR (2012). Serum hepcidin levels and iron parameters in children with iron deficiency. Korean J Hematol.

[42] Jonker FA, Calis JC, Phiri K, Kraaijenhagen RJ, Brabin BJ, Faragher B (2013). Low hepcidin levels in severely anemic malawian children with high incidence of infectious diseases and bone marrow iron deficiency. PLoS One.

[43] Thomas DW, Hinchliffe RF, Briggs C, Macdougall IC, Littlewood T, Cavill I (2013). Guideline for the laboratory diagnosis of functional iron deficiency. Br J Haematol.

[44] Yilmaz H, Cakmak M, Darcin T, Inan O, Bilgic MA, Bavbek N (2016). Can serum Gdf-15 be associated with functional Iron deficiency in hemodialysis patients?. Indian J Hematol Blood Transfusion.

[45] Li XY, Ying J, Li JH, Zhu SL, Li J, Pai P (2015). Growth differentiation factor GDF-15 does not influence iron metabolism in stable chronic haemodialysis patients. Ann Clin Biochem.

[46] Ramirez JM, Schaad O, Durual S, Cossali D, Docquier M, Beris P (2009). Growth differentiation factor 15 production is necessary for normal erythroid differentiation and is increased in refractory anaemia with ring-sideroblasts. Br J Haematol.

[47] Tanno T, Rabel A, Lee YT, Yau YY, Leitman SF, Miller JL (2010). Expression of growth differentiation factor 15 is not elevated in individuals with iron deficiency secondary to volunteer blood donation. Transfusion..

[48] Mast AE, Foster TM, Pinder HL, Beczkiewicz CA, Bellissimo DB, Murphy AT (2008). Behavioral, biochemical, and genetic analysis of iron metabolism in high-intensity blood donors. Transfusion..

[49] Nalado AM, Mahlangu JN, Duarte R, Paget G, Olorunfemi G, Jacobson BF (2018). Utility of reticulocyte haemoglobin content and percentage hypochromic red cells as markers of iron deficiency anaemia among black CKD patients in South Africa. PLoS ONE.

[50] Xiang-Yang Li JY, Li J-H (2014). Growth differentiation factor GDF-15 does not influence iron metabolism in stable chronic haemodialysis patients. Ann Clin Biochem.

[51] Theurl I, Finkenstedt A, Schroll A, Nairz M, Sonnweber T, Bellmann-Weiler R (2010). Growth differentiation factor 15 in anaemia of chronic disease, iron deficiency anaemia and mixed type anaemia. Br J Haematol.

[52] Mei SWH, Fu R (2014). Hepcidin and GDF15 in anemia of multiple myeloma. Int J Hematol.

[53] Omaima M, Abbas MAH, Ashraf Y (2015). El Fert, et al. growth differentiation factor 15 as a marker of ineffective erythropoesis in patients with chronic C virus infection. Menoufia Med J.

[54] De Haan JJ, Haitjema S, den Ruijter HM, Pasterkamp G, de Borst GJ, Teraa M (2017). Growth Differentiation Factor 15 Is Associated With Major Amputation and Mortality in Patients With Peripheral Artery Disease. J Am Heart Assoc.

