# Supplementary material for: Hepcidin and GDF-15 are potential biomarkers of iron deficiency anaemia in chronic kidney disease patients in South Africa
Source: BMC Nephrol. 2020 Sep 29;21:415. doi: 10.1186/s12882-020-02046-7 (PMC7523312; doi:10.1186/s12882-020-02046-7)
Supplement: Supplementary file 1 — Additional file 1 Table S1: Correlations of hepcidin levels with haematological and other parameters. Table S2: Correlation of GDF-15 levels and iron parameters among the study population. Table S3: Cut-off and validity of GDF-15 and Hepcidin in diagnosing Absolute and functional Iron deficiency Anaemia among CKD patients. Table S4. Relationship between hepcidin and GDF15 levels across stages and aetiology of Chronic kidney disease. Table S5. Performance of Hepcidin and GDF-15 in the diagnosis of iron deficiency anaemia among chronic kidney disease participants across stage and aetiology of kidney disease. [file 12882_2020_2046_MOESM1_ESM.doc]

**Supplementary tables**

Supplementary Table 1: Correlations of hepcidin levels with haematological and other parameters

| **Characteristics** | **Hepcidin levels among CKD patients** | | | | **Hepcidin levels among Controls** | | | |
| --- | --- | --- | --- | --- | --- | --- | --- | --- |
|  | **IDA** | | **NIDA** | | **IDA** | | **NIDA** | |
|  | R | **p-value** | r | **p-value** | R | **p-value** | R | **p-value** |
| GDF -15 | -0.28 | 0.0037 | -0.07 | 0.289 | -0.36 | 0.141 | 0.057 | 0.469 |
| Serum Ferritin | 0.50 | <0.0001 | 0.57 | <0.0001 | 0.25 | 0.325 | 0.45 | <0.0001 |
| TSAT | -0.031 | 0.759 | 0.21 | 0.002 | 0.48 | 0.046 | 0.23 | 0.003 |
| Haemoglobin | 0.017 | 0.862 | -0.08 | 0.238 | 0.29 | 0.237 | 0.28 | 0.0003 |
| GFR | -0.19 | 0.049 | -0.17 | 0.012 | -0.20 | 0.418 | -0.26 | 0.006 |
| Age | -0.18 | 0.064 | 0.004 | 0.957 | 0.004 | 0.987 | 0.02 | 0.817 |
| hsCRP | -0.007 | 0.947 | 0.01 | 0.846 | -0.25 | 0.321 | -0.02 | 0.804 |
| MCV | 0.02 | 0.841 | 0.09 | 0.178 | 0.38 | 0.124 | 0.04 | 0.616 |
| Serum Urea | 0.23 | 0.019 | 0.13 | 0.069 | 0.42 | 0.083 | 0.03 | 0.709 |
| Serum Creatinine | 0.21 | 0.037 | 0.17 | 0.016 | 0.30 | 0.234 | 0.25 | 0.001 |
| Serum Albumin | -0.18 | 0.070 | -0.10 | 0.146 | 0.03 | 0.911 | 0.06 | 0.418 |
| MCHC | 0.003 | 0.976 | -0.072 | 0.302 | 0.26 | 0.295 | 0.23 | 0.003 |
| Calcium | -0.21 | 0.034 | -0.068 | 0.325 | 0.23 | 0.350 | 0.13 | 0.0942 |
| Phosphate | 0.26 | 0.008 | 0.04 | 0.546 | -0.008 | 0.976 | -0.26 | <0.0001 |

Abbreviations: IDA iron deficiency anaemia; NIDA: non- iron deficiency anaemia; TSAT: transferrin saturation; hsCRP: highly sensitive C-reactive protein; MCV: mean corpuscular volume; MCHC: mean corpuscular haemoglobin concentration

Supplementary table 2: Correlation of GDF-15 levels and iron parameters among the study population

| **Parameters** | **GDF-15 levels among CKD patients** | | | | **GDF-15 levels among Controls** | | | |
| --- | --- | --- | --- | --- | --- | --- | --- | --- |
|  | **IDA** | | **NIDA** | | **IDA** | | **NIDA** | |
|  | **R** | **p-value** | **R** | **p-value** | **R** | **p-value** | **r** | **p-value** |
| Serum Ferritin | -0.29 | 0.003 | -0.009 | 0.902 | -0.03 | 0.918 | -0.09 | 0.229 |
| TSAT | 0.13 | 0.192 | 0.09 | 0.219 | -0.02 | 0.938 | 0.017 | 0.824 |
| Haemoglobin | -0.10 | 0.314 | -0.34 | <0.0001 | -0.55 | 0.018 | -0.23 | 0.003 |
| eGFR | -0.11 | 0.290 | -0.17 | 0.01 | -0.06 | 0.817 | 0.09 | 0.225 |
| Age | 0.16 | 0.108 | 0.001 | 0.988 | 0.01 | 0.955 | 0.06 | 0.472 |
| hsCRP | -0.03 | 0.772 | 0.08 | 0.247 | -0.04 | 0.876 | -0.13 | 0.100 |
| MCV | -0.16 | 0.110 | -0.08 | 0.223 | -0.42 | 0.086 | -0.04 | 0.573 |
| MCHC | -0.05 | 0.616 | -0.19 | 0.005 | -0.16 | 0.525 | -0.025 | 0.751 |
| Serum Creatinine | 0.10 | 0.336 | 0.16 | 0.025 | 0.19 | 0.440 | -0.11 | 0.173 |
| Serum Albumin | 0.07 | 0.504 | -0.04 | 0.564 | 0.13 | 0.595 | -0.09 | 0.255 |
| Calcium | 0.07 | 0.460 | -0.11 | 0.119 | 0.31 | 0.214 | -0.11 | 0.178 |
| Phosphate | -0.02 | 0.807 | 0.15 | 0.035 | -0.11 | 0.650 | -0.05 | 0.519 |
| Serum Urea | 0.07 | 0.489 | 0.19 | 0.006 | -0.04 | 0.877 | 0.03 | 0.710 |

Abbreviations: IDA: iron deficiency anaemia; NIDA: non- iron deficiency anaemia; TSAT: transferrin saturation, hsCRP: highly sensitive C - reactive protein; MCV: mean corpuscular volume; MCHC: mean corpuscular haemoglobin concentration

Supplementary Table 3 : Cut-off and validity of GDF-15 and Hepcidin in diagnosing Absolute and functional Iron deficiency Anaemia among CKD patients.

| **Test Parameter** | **Predictive value of GDF 15 for AID among CKD patients** | **Predictive value of Hepcidin for FID among CKD patients** |
| --- | --- | --- |
|  | **Value** | **Value** |
| Cut-off | 1129.3 mg/dl | 22.5ng/dl |
| Non-covariate AUC | 74.02 (95% CI: 67.62% - 80.42%) | 70.1 (95% CI: 62.79% - 77.49%) |
| Sensitivity | 83.64% | 66.7% |
| Specificity | 66.03% | 70.8% |
| Likelihood ratio of positive result | 2.462 | 2.28 |
| Likelihood ratio of negative result | 0.2478 | 0.47 |
| Younden’s index | 49.67% | 37.48% |

AID; Absolute iron deficiency anaemia FID; Functional iron deficiency anaemia

AUC; Area Under Curve.

**Supplement Table 4. Relationship between hepcidin and GDF15 levels across stages and aetiology of Chronic kidney disease**

| **Kidney parameters** | **ID** | | **NID** | | **ID** | | **NID** | |
| --- | --- | --- | --- | --- | --- | --- | --- | --- |
|  | **Median(IQR) Hepcidin**  **Level** | **P-value** | **Median (IQR) Hepcidin**  **Level** | **P-value** | **Median (IQR) GDF-15**  **Level** | **P-value** | **Median GDF-15(IQR)**  **Level** | **P-value** |
| **Stages of kidney disease** | | | | | | | | |
| I | 4(3.9 – 4.8) | 0.1820# | 4.3(3.85 – 24.1) | 0.0373# | 1256.8 (1722.6 - 1722.6) | 0.3705 | 604.4(257.05 - 1165.5) | 0.0173 |
| II | 23.2 (6.15 – 30.5 ) |  | 4.15(3.15 – 10.7) |  | 1170.3 (724.05 - 1220.45) |  | 526.55 (288.7 - 1362.9) |  |
| IIIA | 4.2 (4.05 - 187.35) |  | 7.95(4-18.6) |  | 1244.7 (637.85 - 1604.45) |  | 572.6 (317.2 - 1290) |  |
| IIIB | 9.4(3.5 – 33.9) |  | 7.7(4.05 – 43.4) |  | 1183 (442.5 – 1618) |  | 596.8 (263.5 - 1004.65) |  |
| IV | 5.3(3.9 - 44.4) |  | 4.9(3.5 – 23.4) |  | 1389.25(1059 – 1536) |  | 999.3 (532.4 - 1556 |  |
| V | 28.8(4.3 - 79.6) |  | 35.75(11.4 – 62.85) |  | 1298 (701.1 - 1756.8) |  | 1372.15(865.4 - 1756.5) |  |
|  |  |  |  |  |  |  |  |  |
| Early (I-III) | 4.7 (3.9 – 32.55) | 0.2583^$^ | 6.2(3.9 – 21.6) | 0.1387^$^ | 1190.9 (657.05 - 1530.3) | 0.0773 | 590.4(265.4 – 1197) | 0.0007 |
| Late (IV -V) | 10.1 (4.1 - 55.8) |  | 14.3(4 – 48) |  | 1345.8(1052 - 1345.8) |  | 1165 (566.8 – 1578) |  |
| **Aetiology of CKD** | | | | | | | | |
| 1 | 8.3(4 – 39.5) | 0.7793^#^ | 8.85 (4 – 47.9) | 0.1083^#^ | 1244.8 (797.6 – 1487.0) | 0.6215 | 650.55  (337.9-1288 | 0.2939# |
| 2 | 5.8(3.85 - 63.25) |  | 8.85(3.6 – 14.9) |  | 1283.4(1048.5 - 1739.7) |  | 785.2(332.1 - 1459.8) |  |
| 3 | 10.8(4.85 – 41.5) |  | 4.5 (3.2 – 15.1) |  | 1661.4 (831.2 - 1806.95) |  | 456.8 (209.9 – 1066) |  |
| 4 | 36.65(4/1 – 72.1) |  | 10.8 (3.5 – 61.3) |  | 1260.2 (1175.8 – 1572.0) |  | 1100.9(421.2 - 2128) |  |
| ^$^ Mann Whitney U test^; #^ Kruskal Wallis’ test, ID : Iron deficiency anaemia; NID : Non-iron deficiency; IQR :Interquartile range. 1=Hypertension, 2=Diabetes Mellitus, 3= Adult polycystic Kidney Disease, 4= Others | | | | | | | | |

**Supplementary Table 5**. Performance of Hepcidin and GDF-15 in the diagnosis of iron deficiency anaemia among chronic kidney disease participants across stage and aetiology of kidney disease

| **Characteristics** | **AID** | | **FID** | | **ID** | |
| --- | --- | --- | --- | --- | --- | --- |
|  | **Hepcidin** | **GDF15** | **Hepcidin** | **GDF15** | **Hepcidin** | **GDF15** |
|  | **AUC,ROC** | **AUC,ROC** | **AUC,ROC** | **AUC,ROC** | **AUC,ROC** | **AUC,ROC** |
| **Stage of disease** | | | | | | |
| **Early (I-III)** | NS | 0.8325 | NS | NS | NS | 0.7747 |
| **Late (IV- V)** | NS | NS | 0.7523 | NS | NS | NS |
| **Aetiology of CKD** | | | | | | |
| **1** | NS | 0.8106 | NS | NS | NS | 0.8187 |
| **2** | NS | NS | 0.8516 | NS | 0.7964 | NS |
| **3** | NS | NS | NS | NS | NS | NS |
| **4** | NS | NS | NS | NS | NS | NS |
|  |  |  |  |  |  |  |
| **All CKD participants** | NS | 0.7785* | 0.7963* | NS | 0.7701* | 0.7727* |
| AID: Absolute Iron deficiency anaemia; FID: Functional Iron deficiency anaemia; ID: Iron deficiency anaemia; 1= Hypertension; 2= Diabetes Mellitus; 3= Adult Polycystic Kidney Disease ; 4=Others.  NS: non-significant relationship between Hepcidin or GDF -15 and anaemia types after multivariable regression modelling  AUC, ROC: Area under the Receiver operator characteristics curve  *ROC value of multivariable Model of association between hepcidin or GDF-15 and anaemia type, corrected for Age, gender, CKD stage, history of Diabetes, race, C-reactive protein and Mean Corpuscular Volume. | | | | | | |
